# Baby-led weaning: what a systematic review of the literature adds on

**DOI:** 10.1186/s13052-018-0487-8

**Published:** 2018-05-03

**Authors:** Enza D’Auria, Marcello Bergamini, Annamaria Staiano, Giuseppe Banderali, Erica Pendezza, Francesca Penagini, Gian Vincenzo Zuccotti, Diego Giampietro Peroni, on behalf of the Italian Society of Pediatrics

**Affiliations:** 1 Department of Pediatrics, Ospedale dei Bambini V. Buzzi, University of Milan, Milan, Italy; 2 Department of Medical Sciences, University of Ferrara, Ferrara, Italy; 3 Dipartimento di Scienze Mediche Traslazionali, Università degli Studi di Napoli Federico II, Naples, Italy; 4 Department of Pediatrics ASST Santi Paolo e Carlo Hospital, Department of Health Science, University of Milan, Milan, Italy; 5 Department of Clinical and Experimental Medicine, Section of Paediatrics, University of Pisa, Pisa, Italy

## Abstract

The term weaning describes the time period in which a progressive reduction of breastfeeding or the feeding of infant-formula takes place while the infant is gradually introduced to solid foods. It is a crucial time in an infant’s life as not only does it involve with a great deal of rapid change for the child, but it is also associated with the development of food preferences, eating behaviours and body weight in childhood and also in adolescence and adulthood.

Therefore, how a child is weaned may have an influence later, on the individual’s entire life. Babies are traditionally first introduced to solid foods using spoon-feeding, in most countries.

Beside to traditional approach, an alternative method, promoting infant self-feeding from six months of age, called baby-led weaning or “auto-weaning”, has grown in popularity. This approach causes concern to healthy professionals and parents themselves as data from observational studies pointed out to a potential risk of iron and energy inadequacy as well as choking risk. Aim of this systematic review was to critically examine the current evidence about baby-led weaning approach and to explore the need for future research.

A systematic search was conducted in Cochrane library databases and DARE (Database of Abstract of Reviews of Effects), EMBASE and MEDLINE in the period 2000–2018 (up to March 1st) to address some key questions on baby-led weaning. Prisma guidelines for systematic reviews has been followed.

After the inclusion/exclusion process, we included for analysis of evidence 12 articles, 10 observational cross-sectional studies and 2 randomized controlled trials. Pooling of results from very different outcomes in the studies included was not possible. Both randomized trials have potential bias; therefore, the quality of the evidence is low.

There are still major unresolved issues about baby-led weaning that require answers from research and that should be considered when advices are requested from health professionals by parents willing to approach this method.

## Background

The term weaning describes the time period in which a progressive reduction of breast-feeding or the feeding of infant-formula takes place while the infant is gradually introduced to complementary foods. The introduction of complementary foods during the weaning period is generally progressive, and leads the infant to reach the dietary pattern of an adult within the second year of life [1].

The World Health Organization (WHO) recommends exclusive breastfeeding for the first six months of age, and complementary breastfeeding at least until the second year of age. According to the WHO, the introduction of complementary foods should be safe, well-timed and adequate; it should start when exclusive breastfeeding can no longer provide enough nutrients and energy for the infant’s growth and development, and it should contain foods that offer these nutrients and energy [2]. The weaning period is a crucial time in an infant’s life since it not only involves a great deal of rapid change for the child, but it is also associated with the development of food preferences, eating behaviours and body weight in childhood, adolescence as well as in adulthood.

Babies are traditionally first introduced to solid foods using spoon-feeding of specially prepared thin purées. Later, following the infant’s age and developmental progress, the foods offered gradually shift towards family foods [3].

Over the last 10–15 years, an alternative approach known as “baby-led weaning” (BLW), or “auto-weaning”, has grown in popularity, particularly in the United Kingdom and New Zealand and more recently also in other countries in Europe [4, 5]. The term “baby-led” weaning was first coined by Gill Rapley in 2005 [6]. BLW is an alternative method of infant feeding which promotes infant self-feeding from six months of age, instead of conventional parent spoon-feeding. Although BLW is not especially mentioned in the WHO’s recommendations, it is becoming more popular. The key features of BLW are that infants participate in family mealtimes, and ‘whole’ (baby-fist size) pieces of food are offered to them, so they feed themselves from the beginning of complementary feeding, at around six months of age [7]. It means that although parents offer foods, the child himself controls the weaning process (thus the term “baby-led”): infants decide what, how much and how quickly to eat [8]. The term underlines the fact that the infant is an active partner in the feeding process, and not a passive recipient to fill with food [9].

BLW may also be defined as auto-weaning, which means offering chopped and minced family meals to the infants [10]. While in the traditional weaning infants are offered puréed infant foods that are often made up of several ingredients, in the baby-led weaning a variety of single picked foods is offered to the baby. In the former approach, the tastes of the single foods are mixed together and the child is not always able to distinguish them; conversely, BLW approach might provide an early and more stable learning about the satiating capacities of foods and therefore it may enable a better satiety-responsiveness [11].

BLW seems to be associated with other positive aspects, such as lower maternal anxiety and control during the weaning period, but this point is controversial [12].

A recent review has found that many mothers use food to influence infant growth, contentment and sleep and that they choose ease of feeding over infant feeding recommendations [13].

However, this approach also causes concerns in primary care paediatricians and in parents themselves, regarding main topics such as iron adequacy [14, 15], energy and nutrient intake and choking risk [10].

The European Society for Paediatric Gastroenterology, Hepatology and Nutrition (ESPGHAN 2017), in a recent position paper, stated that there is not enough evidence to draw conclusions about the BLW approach [16]. At the same time, a non-systematic review by Brown et al., considering the available literature on BLW up to December 2016, came to similar conclusions [4].

### Aim

The aim of this systematic review was to critically examine the current evidence about the BLW approach, in order to assess whether it is safe and advisable for parents and infants as well as to explore the need for future research.

## Methods

We aimed to address the following Key Questions**:**

1. Does BLW increase risk of choking?
2. Does BLW determine adequate energy intake and normal growth?
3. Does BLW cause an increased risk of inadequate iron intake and resulting suboptimal iron status?
4. Which effects has the BLW approach on satiety-responsiveness and weight?
5. Does BLW influence food preferences and diet quality?
6. Does BLW improve family relationships during shared meals?
7. Do mothers who adopt a baby-led approach differ from those who choose traditional weaning regarding the starting time of complementary feeding?
8. Does BLW have positive effects on mother anxiety and attitude towards complementary feeding?

To answer these Key Questions we searched, from 2000 up to March 1, 2018 the Cochrane Database of Systematic Reviews and DARE (Database of Abstract of Reviews of Effects), for terms “*weaning*” and “*baby-led weaning*”.

The details of search strategies in PubMed (MedLine) and EMBASE are listed in Table 1.

**Table 1:** Details of search strategies in PubMed (MedLine) and EMBASE

PubMed	- “baby led weaning” OR “baby led weaning choking” OR “self-weaning” ((“infant, newborn”[MeSH Terms] OR (“infant”[All Fields] AND “newborn”[All Fields]) OR “newborn infant”[All Fields] OR “baby”[All Fields] OR “infant”[MeSH Terms] OR “infant”[All Fields]) AND led[All Fields] AND (“weaning”[MeSH Terms] OR “weaning”[All Fields]) AND (“airway obstruction”[MeSH Terms] OR (“airway”[All Fields] AND “obstruction”[All Fields]) OR “airway obstruction”[All Fields] OR “choking”[All Fields])) OR ((“infant, newborn”[MeSH Terms] OR (“infant”[All Fields] AND “newborn”[All Fields]) OR “newborn infant”[All Fields] OR “baby”[All Fields] OR “infant”[MeSH Terms] OR “infant”[All Fields]) AND led[All Fields] AND (“weaning”[MeSH Terms] OR “weaning”[All Fields])) OR “self-weaning”[All Fields] AND (“2000/01/01”[PDAT] “March 1, 2018”[PDAT])
EMBASE	- “baby led weaning” OR “baby-led weaning” OR “self-weaning” OR “autoweaning” ((‘baby led weaning’/exp. OR ‘baby led weaning’) OR (‘baby led’ AND (‘weaning’/exp. OR weaning)) OR (‘self weaning’) OR (autoweaning)) AND [2000–2018]/py

We planned hand-search for other relevant articles from selected papers.

Our inclusion criteria, with respect to the predefined aims of the review, were: evidence-based guidelines, systematic reviews, randomized controlled trials, controlled clinical trials and observational studies that compared outcomes related to growth and development, energy, macro- and micronutrient intake and feeding attitudes in children and families that are following BLW approach or standard/traditional complementary feeding; outcomes related to the impact of different weaning styles on mothers feelings and attitudes about their children are considered.

No exclusions were made for age nor restriction for language.

Practice guidelines not evidence-based, narrative reviews, editorials, other publication types and articles in which BLW was not clearly defined and/or quantified, were considered for discussion of items but excluded from formal analysis and from tables of evidence.

We planned to appraise the relevant guidelines with the AGREE II instrument [17], systematic reviews with the AMSTAR-2 tool (A MeaSurement Tool to Assess systematic Reviews) [18], Randomized Controlled Trials with the Assessment of Risk of Bias Cochrane tool [19], Observational studies with the Newcastle-Ottawa Scale, modified for cross-sectional studies [20].

ED and MB independently searched and selected the literature based on the inclusion/exclusion criteria, then extracted results and appraised the included articles.

We considered methodological lacks of each study to answer Key Questions.

We also planned the pooling of results, with appropriate methods, if the same outcome was present in a sufficient number of not heterogeneous studies.

## Results

After the inclusion/exclusion process, we included 12 articles, 10 observational cross-sectional studies and 2RCT (from the same population) for analysis of evidence.

**Fig. 1:**
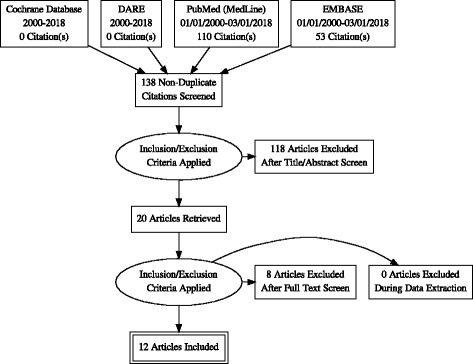
PRISMA Flow Diagram

In Fig. 1 we show the PRISMA Flow Diagram [21] of search and selection.

The characteristics of the included studies are listed in Table 2 and described in the text answers to Key Questions.

**Table 2:** Characteristics of included studies and main results

Reference	Type of study	Type of data collection	Number of subjects	Age of infants	Method of weaningconsidered	Definition of baby-led weaning	Intervention	Outcomes considered
Brown and Lee, 2011 [30]	Observational (comparative cross-sectional)	Online questionnaire (self- reported)	*N* = 655 mothers	6–12 months	BLW vs traditional spoon-feeding	BLW = 10% or less of puréed foods and spoon-feeding (self-reported)	/	Demographic background of mothers, timing and type of weaning, experiences of introducing solid foods to infants.
Brown and Lee, 2011 [11]	Observational (comparative cross-sectional)	Child Feeding Questionnaire (self-reported)	*N* = 702 mothers	6–12 months	BLW vs traditional spoon-feeding	BLW = using both spoon feeding and purées 10% or less (self-reported)	/	Weaning approach, Information regarding infant weight, perceived size and mothers’ level of control.
Townsend and Pitchford, 2012 [24]	Observational (comparative cross-sectional on current and retrospective data)	Self-completed questionnaire	*N* = 155 parents	20–78 months	baby-led weaning vs traditional spoon feeding	Self-reported weaning style	/	Impact of the weaning methods on food preferences and health-related outcomes (BMI)
Cameron et al., 2013 [25]	Observational (comparative cross-sectional)	Online survey	*N* = 199 mothers	6–7 months	BLW vs traditional spoon-feeding	adherent BLW = infant mostly or entirely self-feeding; self-identified BLW = mothers reporting following BLW but using at least 50% spoon-feeding;parent-led feeding= > 50% spoon-feeding	/	Comparison between the different feeding practices and selected health-related behaviours (timing and type of complementary food, mealtimes, choking, demographic information)
Moore et al., 2014 [38]	Observational (comparative cross-sectional)	Parental online questionnaire	*N* = 3207 parents	17–26 weeks	All	Self-defined	/	Factors associated with timing of weaning
Brown and Lee, 2013 [12]	Observational (comparative cross-sectional)	self-report questionnaire	*N* = 298 mothers	18–24 months	BLW vs traditional approach	BLW = 10% or less of puréed foods and spoon-feeding	/	Maternal demographic information, child eating style (satiety-responsiveness, food-responsiveness, fussiness, enjoyment of food) and reported child weight and BMI.
Brown, 2016 [40]	Observational (comparative cross-sectional)	Maternal self-reported questionnaire, including Dutch Eating Behaviour Questionnaire, Brief Symptom Inventory and Ten Item Personality Questionnaire	*N* = 604 mothers	6–12 months	BLW vs traditional approach	BLW = 10% or less of puréed foods and spoon-feeding	/	Maternal characteristics and demographic background, weaning style, maternal personality and eating behavior
Brown, 2017 [26]	Observational (comparative cross-sectional)	Maternal self-reported questionnaire	*N* = 1151 mothers	4–12 months	BLW (strict or loose) vs traditional approach	Self-reported strict or loose BLW or traditional approach; estimated frequency of spoon-feeding (0, 10, 50, 75, 90, 100%)	/	Comparison of number of choking episodes, type of foods offered- > No significant differences in choking episodes between groups
Cameron et al., 2015 [29]	Observational (comparative cross-sectional)	weekly interview for 12 weeks and three-day weighed record or iron questionnaires	*N* = 23 infants(14 BLISS, 9 BLW)	6 months (followed until 9 months)	BLW and BLISS (Baby-Led Introduction to SolidS)	Self-defined BLW or BLISS approach	BLW group: no intervention (no feeding protocol to follow). BLISS group: 2 visits and support about the characteristics of BLISS approach.	Comparison of high energy foods, iron containing foods, high choking risk foods offered. - > the BLISS group was more likely to introduce iron containing foods and less likely to be offered high-choking-risk foods
Morison et al., 2016 [31]	Observational (comparative cross-sectional)	Parental feeding questionnaire and weighed diet record	*N* = 51 infants (25 BLW, 26 traditional spoon-feeding TSF)	6–8 months	Baby-led vs traditional spoon-feeding	Self-defined BLW or traditional approach	/	Comparison of food, nutrient and family meal intakes.- > BLW and TSF infants had similar energy intakes; BLW had higher intakes of fat and saturated fat, and lower intakes of iron, zinc and vitamin B12. Many in of both groups were offered high choking risk foods.
Fangupo et al., 2016 [27]	RCT	Maternal report in 5 questionnaires, 2 daily calendars and 2 weighed diet records	N = 206 healthy women in late pregnancy	Newborn (followed until 12 months)	BLISS vs traditional spoon-feeding	Randomisation to either BLISS or control	Control group: free well child health care, conventional complementary feeding methods. BLISS group: 8 additional parent contacts for education and support regarding the BLISS approach to complementary feeding.	Comparison of choking and gagging- no significant group differences in n° of choking events at any time (BLISS infants gagged more frequently at 6 months but less frequently at 8 months than controls)- 35% of infants choked at least once between 6 and 8 months of age - > a large n° of children in both groups was offered foods that pose a choking risk
Taylor et al., 2017 [32]	RCT	Questionnaires and 3-day weighed diet records	*N* = 206 healthy women in late pregnancy(105 BLISS, 101control) At 24 months, *N* = 166	Newborn (followed until 24 months)	BLISS vs traditional spoon-feeding	Randomisation to either BLISS or control	Control group: free well child health care, conventional complementary feeding methods. BLISS group: 8 additional parent contacts for education and support regarding the BLISS approach to complementary feeding.	Primary outcome: BMI z-score at 12 and 24 months. Secondary outcomes: -energy self-regulation and eating behaviors at 6,12,24 months-energy intake at 7,12, 24 months - ≥ mean BMI z-score was not significantly different at 12 months or at 24 months- > in BLISS infants, less food fussiness and greater enjoyment of food reported at 12 months; lower satiety responsiveness at 24 months. - > no significant differences in energy intake at any point

Trials excluded from formal analysis after full-text screening are listed in Appendix 1, with motivations for exclusion.

Pooling of results from very different outcomes in the studies included was not possible.

### - Does BLW increase risk of choking?

Choking can easily occur in infants learning to eat, because they are moving foods around the mouth, chewing and biting for the first times; at six months, the baby may have not yet developed the oral motor skills required to safely ingest whole foods (such as chewing and swallowing) [10, 22].

There may be a discrepancy between the infant’s apparent ability to self-feed and the real capacity to do so; not all 6-month- old children are developmentally ready to start feeding solids [23].

A small survey found no differences in choking incidence between BLW and traditional weaning groups [24]. In 199 BLW infants, 30% had at least one episode of choking with solid food ingestion (apple). It cannot be excluded that this high rate was caused by parents' difficulties to distinguish choking from gagging [25].

Similar results were found by Brown et al. in a recent observational study on 1151 infants addressing the risk of choking and gagging. The results of the study showed at least one episode of choking had occurred in 11.9% of the strict BLW group, in 15,5% of the loose BLW group and in 11.6% of the traditional weaning group, without significant differences among groups [26].

It should be noted that this study is not a randomized study, and that it considers a self-selected sample of mothers, that could lead to less reliable results.

Likewise, a randomized study by Fangupo et al., specifically designed to address the risk of choking, found no differences in choking episodes between the different weaned groups [27].

Fangupo et al., however, did not consider a classical BLW sample of children, but a modified version of BLW, the BLISS [(Baby-led Introduction to Solids (BLISS)] method, providing written and verbal messages to allow parents to learn how to avoid foods more related to choking risk, such as raw apple and grapes, even associated to fatal choking [24, 28, 29]. Hence, it is possible that unmodified BLW may not have the same effects.

The BLISS method was developed and tested by Cameron et coll in 2015 [29].

They aimed to specifically address the concerns of healthcare professionals towards an inadequacy of iron and energy intake, as well as the risk of choking when following BLW. The BLISS method consists of several essential characteristics including offering foods so that the infant can feed themselves similar to a BLW approach, but additionally the method includes advice to offer one high-iron food at each meal, one high-energy food at each meal and food being prepared suitably according to the infant’s level of development to reduce the risk of choking, as well as avoiding high choking-risk foods [27, 29]. The difference between BLW and BLISS is mainly the level of specificity of the instructions, while the key characteristics remain the same.

The pilot study by Cameron has some important limitations, such as a missing group of traditional weaned infants, no random assignment to the groups and only recruiting parents who already planned to use a baby-led approach before-hand. This means that parents who felt confident about the BLW method would rather assign to the BLW group, while parents who felt they needed extra support would choose to assign to the BLISS group. Despite its limitations, this study firstly introduced a modified version of BLW, in which detailed and written instruction are given to the parents.

### - Does BLW determine increased risk of inadequate energy intake and growth faltering?

The results from the observational study by Townsend et al. reported that more BLW children were classified as significantly underweight, compared to spoon-fed children [24]. Another observational study underlined that mothers following a BLW approach estimated that their babies ate more milk feeds and less solid food compared to those following a traditional weaning; this may provide inadequate nutrient intake for infants from 6 months of age onwards [30]. Recently, Morison et al. reported that even though total energy intake was similar between a BLW and a traditional spoon-fed group of infants, BLW infants appeared to consume more total fat and saturated fat than traditional spoon-fed [31].

In agreement with the observational study by Morison et al., the randomized study by Taylor showed no differences in energy intake between the BLISS group and the control group [32].

Additionally, none of the baby-led children in the sample showed growth faltering.

The discrepancies in results compared to the previous studies may be due to the different study design, or to the fact that those studies used infant weight reported by parents, whereas Taylor et colleagues directly measured the infants’ weight. It is also important to address the fact that the BLISS study participants were encouraged to include a high-energy food at each meal, which may have attenuated the risk of growth faltering.

### - Does BLW cause inadequate iron intake and suboptimal iron status?

From six months onwards, breastfeeding does not provide the infant with enough iron to satisfy requirements; therefore, an increased amount of iron is needed from complementary foods. To address this purpose, iron-fortified infant cereals and commercial meat-based infant foods are generally offered. BLW infants may be at risk of inadequate iron intake as the consistence of these foods makes them difficult for babies to self-feed. Furthermore, most easily graspable foods, such as fruits and vapour cooked vegetables, which are the most commonly introduced during BLW, are known to be generally low in iron [25, 33].

Up to now, this issue has been formally addressed in the BLISS study by Cameron et al. [29].

Subjects in the BLISS group were offered more portions of iron rich foods at six months and had a higher introduction of iron containing foods in the first weeks of introduction of solid foods compared to those in BLW group.

Although there was no statistically significant difference in the amount of iron intake from complementary foods by the BLISS and BLW participants who completed the diet records, it must be considered that the sample size was very small (4 in each group). Noteworthy, none of the eight infants for whom diet record data were available were able to achieve the WHO recommendation for iron intake from complementary foods.

### - Which effects has the BLW approach on satiety-responsiveness and weight?

To date, three studies aimed to evaluate the influence of chosen eating feeding on healthy-related outcome, such as body mass index (BMI) and obesity [11, 24, 34]. Townsend and Pitchford found a higher occurrence of underweight children in the baby-led group (3/63) and an increased incidence of obesity in the spoon-fed group (8/63); however, it should be pointed out that 32% of the data on BMI was missing in the baby-led group [24].

In another larger, cross-sectional study Brown and Lee found no association between the weaning approach (BLW vs spoon feeding) and parentally reported infant weight at six months of age [34].

A subgroup of participants in this study were then evaluated at 18–24 months; the aim of this self-reported observational, comparative study was to examine the effect of different weaning practice on child satiety-responsiveness and weight at 18–24 months [11]. Children who were introduced to solids on a BLW approach were reported to be significantly less food responsive, less fussy and more satiety-responsive compared to the traditional weaning group. The authors found that toddlers who had followed BLW had lower mean body weight than the spoon-feeding approach. Again, these findings need to be treated with caution, because the weight was self-reported and the overall number of children in an overweight-range was small in this study. Furthermore, a lot of differences have been shown among parents who followed BLW and traditional weaning, which can influence weight.

The same outcomes were evaluated in a recent randomized clinical trial by Taylor and colleagues [32]. Differently from the previous studies, they found no significant differences in BMI at 12 and 24 months between the baby-led group and the traditionally fed one. In contrast with Brown and Lee, baby-led infants resulted to be less satiety responsive at 24 months of age. Also the study by Taylor, however, did not consider a classical BLW sample of children, but a BLISS one. It is possible that unmodified BLW may not have the same effects as this case.

### - Does BLW influence food preferences and diet quality?

It has been hypothesized that BLW may promote acceptance of a wider range of food as a result of different tastes and textures from the variety of offered foods [33]. This aspect has been formally addressed in two observational studies.Morison BJ et al. found no differences in food preferences between BLW and traditional weaned infants [31]. Conversely, Townsend et al. observed that BLW weaned children had a preference for carbohydrates, whereas the spoon-fed infants preferred sweets [24]. These results require caution as this observational study was based on a retrospective recall of a weaning approach.

Another relevant issue regards the quality of the meals consumed by BLW infants.

Commercial infant foods or home-prepared purées do not usually include sugar and salt. On the other hand, family foods may not always be suitable for infants, specifically regarding the mode of cooking and dressing. For instance, if the family usually consume processed foods or salty foods (i.e, foods flavoured with salt, stock cubes or salad dressing) or snacks, sweets and cereal bars, that infant is likely to be offered it too.

With regard to this issue, the BLISS Study showed that BLISS group consumed higher levels of sodium [35].

Concerns have been expressed that, eating foods which are inappropriate for infants, BLW babies might become accustomed to sugar and salt tastes [22], potentially resulting in increased consumption, which may in turn influence some health outcomes (e.g blood pressure) already during childhood [36].

To avoid these risks, parents should receive a proper nutritional education to make their diet healthy and adequate for the infant.

Further studies are needed to specifically address the impact of baby-led weaning on food preferences and choices during childhood and later health outcomes.

### - Does the baby-led approach improve family relationships during shared meals?

It has been proposed that BLW children may participate in mealtimes more easily than traditional spoon-fed children, because they eat the same foods with the rest of the family. At the same time, as BLW infants are never forced to eat food, there may be less mealtime pressure and anxiety. However, a single non-comparative cross-sectional study found that BLW does not improve the family’s eating style [37].

### - Do mothers who adopt a baby-led approach differ from those who choose traditional weaning regarding the starting time of complementary feeding?

It is not clear if BLW leads to starting complementary feeding later, or if parents who manage to wait until the sixth month of age adopt a BLW approach.

According to the data from observational studies, mothers choosing to follow a baby-led approach appear more likely to begin complementary feeding at six months of age [26, 30, 38]. Data derived from randomized trial are lacking.

As there is some evidence that introduction to solids before six months may increase the risk of overweight in childhood [39], further research is warranted to address this issue.

### - Does BLW have positive effects on mother anxiety and attitude towards complementary feeding?

Baby-led mothers reported lower anxiety, lower obsessive-compulsive disorder scores, lower eating restraints and higher conscientiousness than traditional weaning mothers as shown by the observational study by Brown et al. [40]. These maternal characteristics might make a BLW approach more feasible; however, this finding may also be explained by a reverse causality, according to the fact that mothers high in anxiety may be more likely to choose a traditional weaning approach, where there is more existing literature and support from healthcare professionals, and where the infant’s intake is strictly controlled via spoon-feeding.

## Discussion

One of the critical aspects of the BLW approach is that no formal definition exists. In its purest form, BLW should not include any spoon-feeding and the child himself should put foods into his mouth. As a limitation, most existing studies on the baby-led approach include participant families who self-identify as following a BLW. In some studies, participants were asked to estimate the use of spoon-feeding opposed to self-feeding and the amount of puréed foods given during the weaning period in percent [11, 12, 30, 34]. In others, they were just asked to identify themselves as followers of BLW approach [14, 24, 31]. It is unclear whether BLW can include limited use (less than 10%) of purées and spoon-feeding, or if it is ruled by a more strict definition, where exclusively finger foods are provided. Actually, both views exist among parents who believe in a baby-led style infant feeding [33]. Concerning the method of recruitment, most studies recruited via internet sites, that can represent a selection bias [41]. A recent research conducted in the UK found that mothers following a baby-led approach were more likely to have higher levels of education, and a professional occupation, compared to mothers choosing traditional weaning. This may be due to the fact that the population group with higher education is more likely to have increased internet access, which is also one of the main information sources about BLW [25, 30].

Overall, the evidence about BLW is mostly derived from observational studies, of which only comparative cross-sectional studies were formally analyzed. The methodological quality of the included studies is generally very low, except for two studies, (Table 3).

**Table 3:** Quality assessment scores of selected comparative studies, with Newcastle-Ottawa Scale (modified for cross-sectional)

STUDY	SELECTION (maximum 5 Stars)	COMPARABILITY (maximum 2 Stars)	OUTCOME ASSESSMENT (maximum 3 Stars)	TOTAL (maximum 10 Stars)
Brown et al. 2011 [30]	1	0	1	2
Brown et al. 2011 [11]	1	1	1	3
Townsend et al. 2012 [24]	1	0	1	2
Cameron et al. 2013 [25]	3	2	1	6
Moore et al., 2014 [38]	1	1	1	3
Brown et al. 2013 [12]	2	2	1	5
Brown 2016 [40]	1	2	1	4
Cameron et al. 2015 [29]	2	0	1	3
Morison et al. 2016 [31]	1	0	1	2
Brown et al. 2017 [26]	1	2	1	4

Therefore, the observed results need to be treated with caution. As regards the major concerns about the BLW approach, e.g. inadequate energy intake and choking risk, there are very few data on the role of the paediatrician’s support on this practice. Although the randomized studies by Taylor and Fangupo specifically addressed these issues, in the same cohort, both of them have methodological weaknesses that call into questions the results (Table 4).

**Table 4:** Assessment of risk of bias in RCT (from BLISS population)

Study (outcomes)	Randomization	Allocation concealment	Blinding of participants	Blinding of personnel	Blinding of assessors	Follow-up	Selective reporting	Other
Fangupo et al. 2016 (risk of choking) [27]	Low risk	High risk	High risk	Low risk	High risk	High risk (loss 12% and 15.5% at 6 and 11 months; ITT not performed)	Low risk	Sample size not defined for primary outcome. Outcomes self-reported
Taylor et al. 2017 (BMI, eating behavior, energy intake) [32]	Low risk	High risk	High risk	Low risk	High risk	High risk (loss 14% and 21.5% at 12 and 24 months; ITT not performed)	Low risk (but only few secondary outcomes reported from the original protocol)	Self-reporting of secondary outcomes

Most of the outcomes were self-reported, the intention to treat analysis was not performed and both had a high rate of drop-out. Moreover, the evaluation of nutrient intakes has not been included in the secondary outcomes of the study, but has been described only in an unpublished doctoral dissertation by Erickson, that examines initial findings of the BLISS Study, considering adherence to the weaning approach and nutrient intake, respectively [35]. Therefore, this issue needs a careful monitoring by the paediatrician in order to ensure adequate growth.

Even with the above mentioned limitations, the BLISS Study suggests that this kind of approach, employing resources and methods to educate participants, may be suitable to be tested in further randomized controlled trials.

Anyway, parents willing to follow BLW approach need a careful and deeper nutritional education in order to avoid any risk to their infants. In particular, they should be given advice on how to prepare foods in such a way they result safe, healthy and nutritious.

The role of different approaches to weaning in the development of the early-life gut microbiome is an additional interesting aspect, which has not yet been investigated. It is known that changes in the diet composition can alter the prevalence and types of microbial species living in the gut, as certain species are better equipped to use specific substrates [42].

The first solid foods introduced could therefore play a key role in shaping the infant’s gut microbiome. A recent study, assessing the effect of iron supplementation in infants, found that children fed iron-only fortified cereals, or puréed meats, iron- and zinc-fortified cereals, as the primary complementary foods until 9–10 months of age, had different prevalence in the gut microbial species [43]. The former had a relative decrease with time in the genera *Bifidobacterium* and *Rothia* and in the order *Lactobacillales*, and an abundance of the order *Bacteroidales,* compared to the latter. Further investigations are needed to determine if the BLW approach could shape the microbiome in a different way compared to the traditional weaning.

Another relevant issue is that in the BLW approach children are exposed to a wide range of family foods in a relatively early and mixed manner and sometimes also to packaged foods, which can contain multiple food allergens. Therefore, in case of reaction, it can be difficult to identify the specific food allergen involved.

This potential risk has not been formally investigated, even though the weaning period requires special attention, particularly in children at high risk of allergy.

## Conclusions

Currently, there is still insufficient evidence to draw conclusions about the BLW approach, in terms of adequacy of energy and nutrient intakes, due to the low quality of the evidence. In fact, concerns persist since some previous observational studies indicated that mothers using the BLW approach estimated that their babies ate more milk feeds and less solid foods compared to those following a traditional weaning, focussing attention on inadequate nutrient intakes for infants from 6 months of age onwards. Nevertheless, other evidence from more recent randomized studies suggest that a modified BLW approach (BLISS Study), including recommendations about the introduction of selected iron-rich foods, as well as avoiding foods at risk of choking, might have positive preventive effects on the risk of choking and nutrients deficiency. Thus, these issues require further investigation in larger randomized studies.

In summary, there are still major unresolved issues in BLW that require answers from research, which should be considered when advices are requested from health professionals by parents willing to follow the BLW approach (Table 5).

**Table 5:** Major unresolved issues in BLW and practical advices

Major unresolved issues in BLW (and requirements for further research):	• To assess safety, benefits and potential implications of a baby-led approach in terms of nutrient intakes and baby growth and the risk of choking. • To provide a more standardized definition of BLW, to better compare this approach with a traditional spoon-feeding one. • To perform an accurate quantification of energy and nutrient intakes, by researchers and family doctors themselves, to avoid the potential bias of self-reporting. • To investigate biomarkers, such as biochemical iron, vitamins or oligo-elements, to better assess if the nutritional status of the infants is adequate. • To explore the short-term and long-term impact of BLW on healthy-related outcomes, such as the correct development but also the risks for under- or over-weight, obesity in larger, randomized trials • To investigate whether BLW approach increases or not the risk of food allergic sensitizations and reactions.
Practical advices for parents willing to follow BLW approach:	• To wait until the baby is ready: healthy infants over 6 months of age are developmentally able to self-feed; however, strong chewing skills in some children may not be fully developed until 9 months. • To inform and discuss with the family paediatrician about the approach considering both risks and possible advantages • To monitor with the paediatrician the growth parameters, especially during the first months of weaning and evaluating if supplementations are necessary (i.e. iron, vitamins, oligo-elements ..). • The foods offered should be prepared to be picked up and easily held. • Parents should be advised to avoid added salt and sugar • Meals should be cooked from scratch, without any processed foods. Cooking should be appropriate, i.e., cooking until soft • To include high-iron food, like small pieces of red meat. • To choice a variety of foods, that should gradually be introduced in a broader variety of textures, colours and shapes. • To avoid hard foods, especially small and roundly shaped like nuts and grapes due to the risk of choking. • To pay attention to the infant’s hunger and satiety cues and respond promptly. • To ensure that the child should never be left alone with foods.

## Appendix

**Table 6:** Studies excluded from analysis, whit motivations

Wright et al. 2011	BLW approach not defined
Rowan et al. 2012	Non-comparative cross-sectional study
Cameron et al. 2012	Non-comparative cross-sectional study
Brown et al. 2013	Non-comparative cross-sectional study
Arden et al. 2015	Non-comparative cross-sectional study
Daniels et al. 2016	Congress communication
D’Andrea 2016	Non-comparative cross-sectional study
Daniels et al. 2017	Congress communication
